# Giant basal cell carcinoma in a non-sun exposed area with four years follow-up: a case report

**DOI:** 10.11604/pamj.2024.48.70.44097

**Published:** 2024-06-27

**Authors:** Ghadah Alhetheli

**Affiliations:** 1 Department of Dermatology and Cutaneous Surgery, College of Medicine, Qassim University, Buraidah, Saudi Arabia

**Keywords:** Giant basal cell carcinoma, rare sites, surgical management, prognosis, case report

## Abstract

Basal cell carcinoma (BCC) is a low-grade malignant tumor that if properly managed has an excellent prognosis. Development of BCC outside the skin areas exposed to sun rays is infrequent. Giant BCC is a rare entity, especially in unexposed areas of the body. It carries a considerable implication on patients' quality of life because of the risk of being a source of infection that may progress to severe sepsis and/or metastasis. An old female presented with a long-standing solitary lesion measuring 7.5x6 cm overlaying the lumber 4-5 vertebral region about 2.5 cm from the line of the sacral promontory. No lymph nodes or distant metastases were detected. For many years, it was misdiagnosed by other physicians as eczema, psoriasis, and fungal infection and accordingly failed to respond to treatment. A histopathological examination of lesional punch biopsy assured the diagnosis of giant superficially spreading BCC. The lesion was excised with a circumferential safety margin of about 5 mm. During the follow-up period of 4 years, no recurrence was detected. Despite being a long-standing Giant basal cell carcinoma (GBCC) in a sun-hidden skin area, the growth behaved as a locally malignant lesion without metastasizing. A wide local surgical excision of the GBCC with 5 mm safety margins was feasible, safe, and with a good aesthetic outcome. Importantly, family practitioners should avoid such missed cases through accuracy in their diagnosis and early referral of any atypical cases to a dermatologist.

## Introduction

Basal cell carcinoma (BCC) is the most common malignancy in Caucasian populations. It is characterized by a very low risk of metastasizing and an excellent prognosis if appropriately managed [1]. Most commonly, a BCC is small and located in skin areas that are typically exposed to the sun´s ultraviolet rays. Rarely, does a BCC attain a large size of up to or greater than 5 cm in its greatest dimension; such lesions are termed giant BCCs (GBCCs). GBCCs comprise only about 0.5% of all BCCs, and if they grow to more than 20 cm in surface area are termed super-giant BCCs [2]. Giant BCCs have relevant implications on patients' quality of life [2]. This is because it carries the risk of being a source of infection that may progress to severe sepsis or that induces septic shock and/or carries the risk of metastasizing [1]. Moreover, surgical management of giant BCCs is tricky, and the reconstructive procedures are challenging due to a possible limitation of tissues available for flap coverage [3]. Like in this rare case, the development of BCC outside the areas of ultraviolet exposure, in addition to the atypical presenting manifestations and consequently misdiagnosis of the lesion, are all considerable factors increasing the challenge during surgical management. Hence, we sought to emphasize the importance of early detection of any rare and atypical presentation of GBCC.

## Patient and observation

**Patient information:** a lady in her sixth decade, medically free, presented to the outpatient dermatology clinic in King Fahad Specialist Hospital, Qassim, Saudi Arabia, with a four-year history of skin rash over her lower back. It was previously treated by other practitioners as eczema, psoriasis, and fungal infection but with no response. The patient reported that, over time, the rash had increased markedly in size with occasional itching and mild pain. The patient could not confirm whether the lesion had raised de novo or had developed on top of a pre-existing mole.

**Figure 1:**
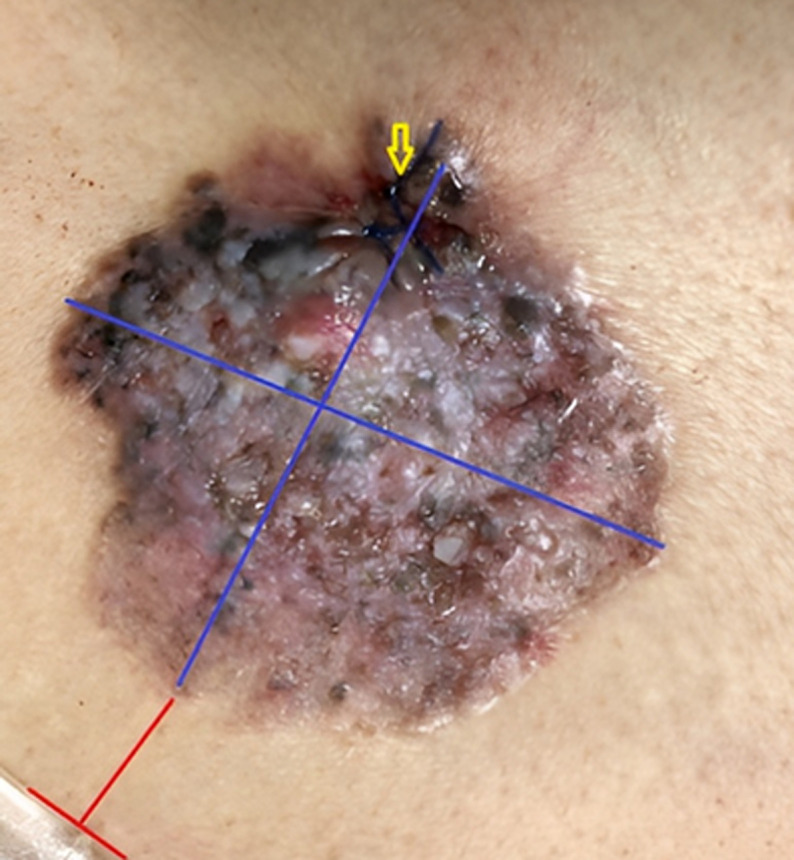
preoperative appearance and dimensions (blue lines) of the lesion with a reference to its location concerning the sacral promontory (red lines); yellow arrow indicates biopsy site

**Clinical findings:** by clinical examination, a solitary lesion measuring 7.5x6 cm in its greatest dimensions (blue lines) was located in the midline overlying the L4-5 vertebral region about 2.5 cm from the line of the sacral promontory (red lines), as shown in (Figure 1). The lesion had an irregular border with definite edge and surface irregularities but no areas of necrotic tissue or ulcerations. The lesion's color was mosaic, erythematous to brownish, with a few scattered areas of grayish and blackish discoloration. An examination of the upper back and the natal cleft areas showed no rashes or moles. A general examination was unremarkable, and the results of routine laboratory investigations were within normal acceptable ranges, showing no manifestations of infection.

**Figure 2:**
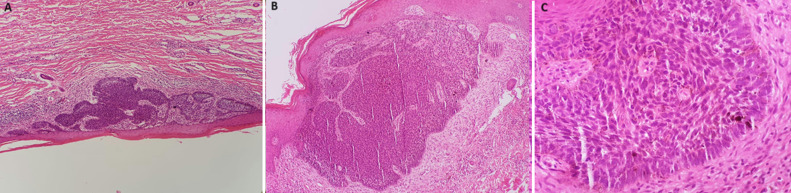
(A) histopathological microscopic appearance of the specimen obtained from the lesion showing a proliferation of basaloid cells parallel to the epidermis; hematoxylin and eosin (H&E) stain; (B) a microscopic examination showed a proliferation of basaloid cells continuous with the epidermis, (H&E) stain; (C) a microscopic examination showed neoplastic palisading cells that are continuous with the epidermis and exhibited scattered mitotic figures, (H&E) stain

**Diagnostic assessment:** a dermoscopic examination wasn´t possible because of the presence of intense inflammation. Considering the location of the lesion, no superficial draining lymph nodes were detected, and fortunately, the PAN-CT scan was unremarkable with no organomegaly or lymph nodal enlargements detected. A punch biopsy of the lesion (4 mm biopsy) was performed (yellow arrow), and a histopathological examination confirmed the diagnosis of a superficially spreading BCC (Figure 2).

**Diagnosis:** the patient was diagnosed with giant superficially spreading BCC.

**Therapeutic interventions:** the patient underwent preoperative preparations and was premedicated with broad-spectrum antibiotics. The surgery was then performed with the patient under general inhalational anesthesia with endotracheal intubation. The lesion was locally excised with a wide circumferential safety margin of 5 mm, and the excision extended deeply to assure the removal of all local spread. A split skin graft was harvested from the inner aspect of the right thigh to close the original defect, and the donor area was allowed to heal by secondary intention. The split-thickness graft was spread to cover the entire original defect and fixed using intradermal interrupted 4-0 vicryl absorbable sutures. Wound drainage was performed using one vacuum drain through the same wound between the interrupted sutures; the drain was removed 48 hours after surgery. The patient was discharged home and returned for follow-up after one week for assurance the wound was healing correctly. Following that visit, she was seen every three months for one year and every six months after to check for any recurrence.

**Follow-up and outcome of interventions:** during the follow-up period of 4 years, no recurrence was detected, and the patient´s complaints disappeared completely.

**Figure 3:**
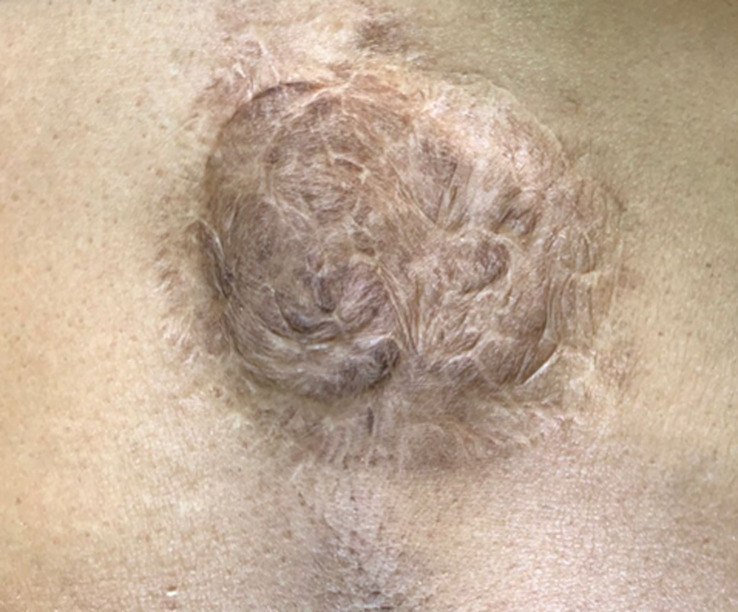
the postoperative appearance of the lesion site at four years follow-up

**Patient perspective:** she was satisfied with the outcome and by the wound´s aesthetic appearance (Figure 3).

**Informed consent:** written informed consent was obtained from the patient for publication of the details of their medical care and any accompanying images.

## Discussion

This study presents the case of a GBCC located in an unusual location at the lower back of a female patient, a site that was previously documented to represent only 10% of BCCs [4]. What makes this case unique is the location of GBCC on the lower back, which is usually an ´absolutely´ sun-covered area in Islamic culture. In addition to that, the atypical presentation had led to long years of being misdiagnosed with many diseases and subsequently receiving treatment that altered the appearance of the lesion without response to medications. Of course, such a diagnosis delay can complicate the management of the case and threaten the patient´s life. It is worth noting that family physicians should avoid such missed cases through accuracy in their diagnosis and early referral of any atypical cases to a dermatologist. The reported location of a BCC outside the skin areas exposed to sun rays indicates other pathogenic mechanisms for the development of the BCC [5].

The therapeutic decision was preoperative preparation with local dressing, anti-inflammatory drugs, and broad-spectrum antibacterial drugs followed by a wide local surgical excision with a safety margin of 5mm surrounding the lesion and a split-thickness skin graft wound closure. No preoperative chemo- or radiotherapy was used to guard against any hazardous side effects, especially in this patient of advanced age. In line with this therapeutic policy, Quéro *et al*. conducted a meta-analysis about treatment policies for skin cancer, and they documented that adjuvant radiotherapy may be used as an alternative to surgery only if surgery is not feasible due to patient´s comorbidities, advanced age or if surgery will end with disfiguring results [6]. A study by Almalki *et al*. described the treatment modalities and outcomes of skin cancer in western Saudi Arabia, finding that surgery was the most common treatment modality and had the highest curative outcome [7]. Hennequin *et al*. suggested that radiotherapy for BCC offered good local control; however, randomized trials indicated the efficacy and efficiency of surgery as a line of management, especially due to its being free of toxicity [8].

Multiple studies have found that GBCC could be lethal secondary to infection and the deterioration of a patient´s immunity, especially when affecting elderly patients, as documented in multiple recent studies [1,2]. However, the current case, fortunately, had no infection but presented with locally inflamed tissue that responded well to preoperative conservative treatment. Surgery was performed through a clean wound site. The above-mentioned studies indicate the importance of preoperative conservative treatment to allow rapid, healthy healing of the wound with satisfactory graft taking. This assumption was evidenced, in the current case, by the short drainage and hospital stay durations, the proper wound healing, and by no graft revision being required. In line with these results, a meta-analysis conducted by Schlager *et al*. found that male gender and immunosuppression were significantly associated with higher infection rates, with an incidence of surgical site infection ranging between 0.96% and 8.70% [9].

During the follow-up period of 4 years, no recurrence was detected. This is in line with the findings reported by Thomson *et al*. who conducted a Cochrane database systemic review which showed that there are fewer recurrence rates with Mohs micrographic surgery (MMS) and surgical excision (SE) at three years follow-up (1.9% versus 2.9%, respectively) with a confidence interval (CI) 0.16 to 2.64. While at 5 five-year follow-ups, MMS and SE have a recurrence rate of 3.2% and 5.2%, respectively, with CI 0.18 to 2.04 [10]. They have also stated in a comparison between MMS and SE that due to the wide 95% CI between these two surgical treatments, there is the possibility of an increase in the risk of recurrence and no differences between the two treatments (low-certainty evidence). Regarding the cosmetic outcomes, they found either little or no differences between MMS and SE [10]. At the end of the follow-up, the patient expressed satisfaction with the outcomes in regard to the disappearance of manifestations, no recurrence, and the aesthetic appearance.

## Conclusion

Despite being a long-standing GBCC in sun-hidden skin areas, the growth behaved as a locally malignant lesion without metastasizing. The wide local surgical excision with 5mm safety margins of the GBCC was feasible and safe, and the aesthetic outcome was satisfactory.
